# Assessing the Amount of Quadruplex Structures Present within G_2_-Tract Synthetic Random-Sequence DNA Libraries

**DOI:** 10.1371/journal.pone.0064131

**Published:** 2013-05-24

**Authors:** Simon A. McManus, Yingfu Li

**Affiliations:** 1 Department of Biochemistry and Biomedical Sciences, McMaster University, Hamilton, Ontario, Canada; 2 Department of Chemistry and Chemical Biology, McMaster University, Hamilton, Ontario, Canada; 3 Michael G. DeGroote Institute for Infectious Disease Research, McMaster University, Hamilton, Ontario, Canada; Florida International University, United States of America

## Abstract

The process of in vitro selection has led to the discovery of many aptamers with potential to be developed into inhibitors and biosensors, but problems in isolating aptamers against certain targets with desired affinity and specificity still remain. One possible improvement is to use libraries enhanced for motifs repeatedly isolated in aptamer molecules. One such frequently observed motif is the two-tiered guanine quadruplex. In this study we investigated whether DNA libraries could be designed to contain a large fraction of molecules capable of folding into two-tiered guanine quadruplexes. Using comprehensive circular dichroism analysis, we found that DNA libraries could be designed to contain a large proportion of sequences that adopt guanine quadruplex structures. Analysis of individual sequences from a small library revealed a mixture of quadruplexes of different topologies providing the diversity desired for an in vitro selection. We also found that primer-binding sites are detrimental to quadruplex formation and devised a method for post-selection amplification of primer-less quadruplex libraries. With the development of guanine quadruplex enriched DNA libraries, it should be possible to improve the chances of isolating aptamers that utilize a quadruplex scaffold and enhance the success of in vitro selection experiments.

## Introduction

Since its development over twenty years ago [1], [2], the process of in vitro selection has led to the isolation of numerous functional nucleic acids, called aptamers, that bind a wide range of target molecules. With their appealing properties, including facile and low cost synthesis, these molecules have great potential to be used for applications such as enzyme inhibition and biosensing. Much research has been carried out to convert aptamers into sensors by coupling their target recognition to diverse signalling platforms, generating many fluorescent, colorimetric, and electrochemical sensors (reviewed in [3], [4]). Despite these advances, the process of in vitro selection that is used to select aptamers remains enigmatic and is in part responsible for hindering aptamers from reaching the mainstream. While sensitive and specific aptamers have been isolated for many targets, in vitro selection experiments have often failed to isolate aptamers for certain targets, or have yielded aptamers that lack the desired affinity or specificity. Currently, in vitro selection is a lengthy process requiring iterative rounds of selection and amplification with no guarantee of success. As such, any means of increasing the chances of isolating potent aptamers by improving an aspect of the current in vitro selection procedure would be of great value.

One possible approach for improving in vitro selection is to design an initial library that in some way increases the number of potential aptamer sequences while maintaining the sequence diversity of the library. It has been observed that some structural motifs arise repeatedly in isolated aptamers and nucleic acid catalysts; the examples include an ATP-binding DNA aptamer [5], [6], the hammerhead ribozyme [7], the 8–17 RNA-cleaving DNAzyme [8]–[12], and a self-phosphorylating DNAzyme [13]. As certain structural motifs seem to be favored, one possible way to enhance the chance of finding aptamers is to pre-design a library with sequences that have a high probability of folding into these structures. One particular motif that arose repeatedly during in vitro selection for DNA aptamers and DNAzymes is the guanine quadruplex motif.

Guanine quadruplexes are four-stranded structures composed of stacks of quartets of guanines [14]–[16]. They are found naturally in the form of telomeric DNA [17] and in the promoter regions of several proto-oncogenes [18]–[21]. Due to their proposed roles in cell immortality and gene regulation these sequence motifs have received much attention as possible cancer-drug targets. These motifs have also been repeatedly identified in functional nucleic acids isolated from numerous in vitro selection experiments. This is particularly true in selections for DNA aptamers and deoxyribozymes [22]–[28]. Some of these quadruplexes have been used to detect targets such as thrombin [29], [30] and potassium [31], [32] and used for drug delivery to cancer cells [33]. The propensity of DNA to form these types of structures may be due to conformational varieties of quadruplexes available to DNA in which the loop residues can be in a number of different arrangements allowing many possible interactions with target molecules by one or more loops. Also noteworthy is that as opposed to the majority of quadruplexes found in biological systems, many of the quadruplexes obtained through in vitro selection contain quadruplexes with only two tiers of guanine quartets [22], [23], [26], [27]. We speculate that this may be due to two-tier quadruplexes being stable enough to provide a structural scaffold, while still being small enough, with only eight bases, to be sampled frequently in a typical random sequence library.

In this study we set out to assess the frequency of two-tiered quadruplex structures within a library containing four pairs of guanines interspersed with random sequences. Studies of this type have been carried out computationally to investigate the types of helical structures present within random sequence libraries [34], and libraries have been designed to include desired amounts of particular helical structural motifs [35]–[37]. Assessing the level of quadruplex formation within a random sequence DNA library by computation methods is more problematic, as folding programs, such as mfold [38], were developed using the free energy of known secondary structures comprised of Watson-Crick base-pairs and do not account for competing structures such as guanine quadruplexes. For quadruplex structural predictions, algorithms do exist to predict the folding and stability of quadruplex-forming sequences [39] but these methods are designed and modelled based primarily on known quadruplexes which are far fewer in number than helical-based structures used for the aforementioned secondary structure prediction algorithms. To investigate whether a sequence will form secondary or tertiary structures, it is necessary to empirically characterize the oligonucleotide using biophysical techniques. This type of analysis has been carried out on G_3_-tract libraries with small partially randomized loops between one and three nucleotides [40] and demonstrated that useful insight can be obtained about the folding and stability of molecules within these libraries. Our analysis will use similar techniques to investigate folding in G_2_-tract libraries. Our study will also look at longer loops that are completely randomized to see whether libraries can be designed that contain sequences folded into quadruplexes despite the large number of randomized nucleotides potentially sequestering G_2_-tracts into other types of structural arrangements.

## Results

### Determining the Parameters for Library Design

We sought to design a DNA library made of four G_2_-tracts interspersed with three random-sequence domains. The four G_2_-tracts would facilitate the formation of the two guanine-quartet tiers of the quadruplex structure and the random regions would function as the connecting loops.

**Figure 1:**
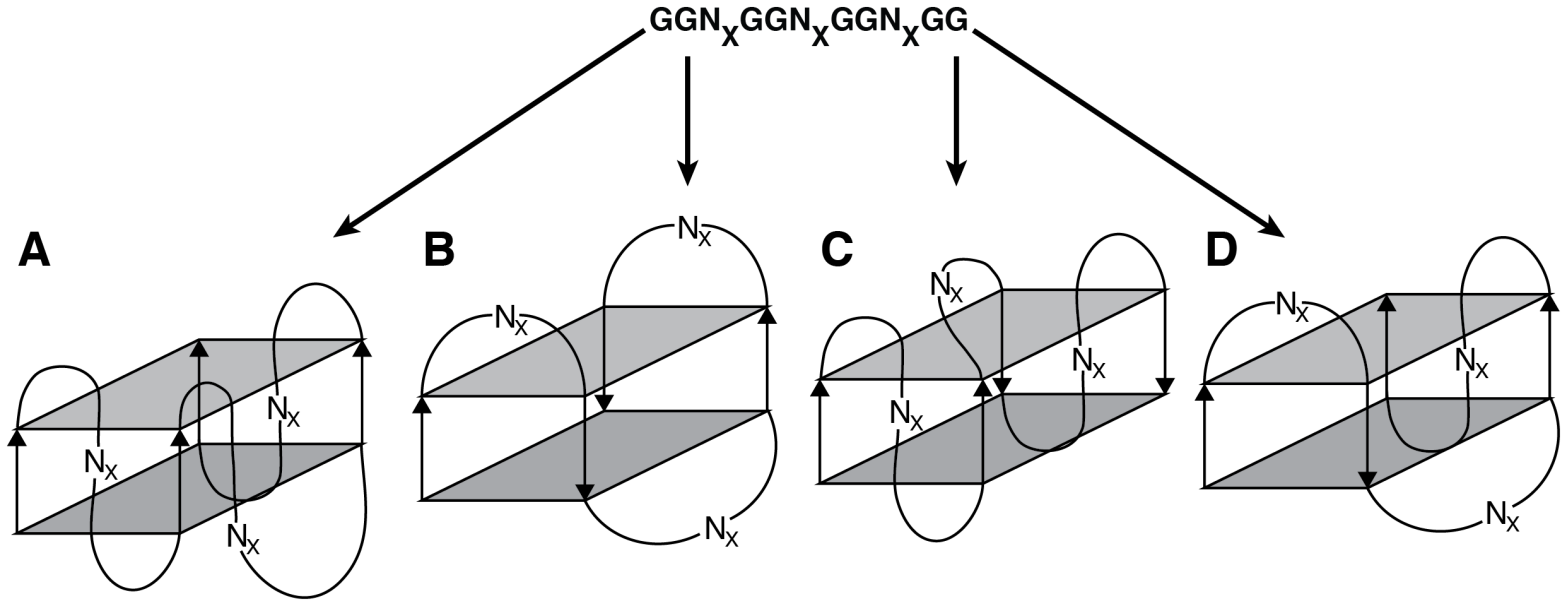
Diversity of potential folding patterns of random-sequence DNA libraries containing 4 G_2_-tracts. Sequences with four GG units and intervening random regions (N_X_) have the potential to form different types of quadruplex structures, such as a quadruplex with three double-chain reversal loops (A), three edgewise loops (B), a diagonal loop, and two double-chain reversal loops (C), or a diagonal loop, and two double-chain reversal loops (D). A listing of all possible topologies can be found in [45].

To determine which loop lengths to test for quadruplex folding and stability, we first considered previous studies on G_3_-tract sequences that may share common features with G_2_-tract sequences. Many G_3_-tract sequences with small loops have been analysed and have been shown to form stable quadruplex structures [41]–[43]. It has also been shown that small changes of sequence in loop regions of certain oligonucleotides has resulted in dramatic changes from one stable quadruplex topology to another [44]. As in vitro selection libraries will contain oligonucleotides with billions and billions of different loop sequences, it is conceivable that many different quadruplex topologies will be represented. With a total of 26 theoretically possible loop orientation topologies for quadruplexes containing three loops [45] (four representative examples are depicted in Figure 1), there is the potential for these libraries to contain a high degree of structural diversity which would be beneficial in an in vitro selection where it is desirable to screen as many different structural variations as possible.

**Figure 2:**
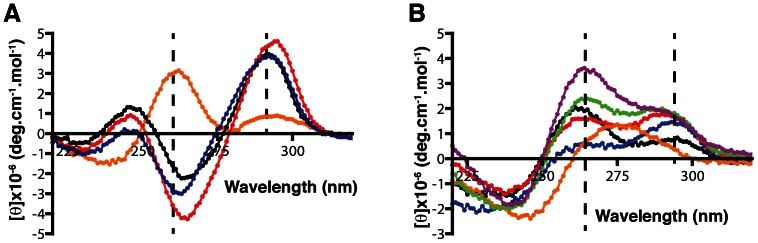
Effects of loop length on G_2_-tract libraries. (A) G_2_-tract sequences (GGT_X_GGT_X_GGT_X_GG) with differing number of intervening thymidine residues were analyzed by circular dichroism between 320 and 220 nm. Series are colored as follows: orange, X = 2; black, X = 3; red, X = 4; blue, X = 5. Dotted lines are placed at 264 and 295 nm as ellipticities at these wavelengths indicate the presence of different subtypes of guanine quadruplexes. (B) G_2_-tract libraries (GGN_X_GGN_X_GGN_X_GG) sequences in which X = 3, 4, 5, 6 and 7 were also scanned; their spectra are shown in black, blue, red, green and purple, respectively. A random sequence of 23 nucleotides was scanned for comparison and the spectrum is shown in orange.

We examined the folding properties of some G_2_-tract sequences with loops containing two or more thymidine residues to confirm whether they share the same folding properties as G_3_-tract sequences. As these previous studies have shown that quadruplexes are typically the most stable in buffers containing K^+^, due to the ionic radius of K^+^ allowing it to fit between two quartet tiers [46], we conducted our analysis of G_2_-tract sequences in a potassium-containing buffer. Circular dichroism (CD) analysis between 320 and 220 nm has been shown to be a powerful technique for the characterization of quadruplex-forming nucleic acids [47] due to quadruplexes being made of overlapping molecular orbitals of stacked guanines that can be differentiated spectroscopically [48]. CD can be used to determine whether DNA sequences are folding into quadruplex arrangements, and even yield information about the topology of the quadruplex formed. Through CD of several quadruplexes of known structure, it has been shown that quadruplexes with different arrangements of glycosidic bond angles of the interacting guanines, resulting in different quadruplex topologies, produce distinct spectra [49]. These quadruplex topologies have been classified into three types. Type I quadruplexes are parallel quadruplexes with all glycosidic bond angles in the *anti* configuration and were found to produce spectra with positive ellipticity around 265 nm and minimal ellipticity at 295 nm. Type II and III quadruplexes are antiparallel quadruplexes, in which the glycosidic bond angles of stacked guanines are in opposite or the same orientation, respectively. Type II quadruplexes show positive ellipticity at 295 nm and contain a second positive ellipticity at 265 nm, giving bimodal spectra. Type III quadruplexes also show positive ellipticity at 295 nm but show negative ellipticity at 265 nm. To test the effect of loop length on the folding of G_2_-tract sequences, we subjected DNA molecules with four pairs of guanines interspaced with different numbers of thymidines (GGT_X_GGT_X_GGT_X_GG) to CD analysis. Sequences will be denoted as G_2_T_x_ with the x representing the number of thymidine nucleotides present in each of the three loops. As shown in Figure 2A, we found that G_2_T_2_ folded into a polymorphic mixture of type I quadruplex structures, as indicated by a large positive peak at 264 nm and a negative peak at 240 nm, as well as type II or III structures indicated by a small positive ellipticity peak at 295 nm. In contrast, G_2_T_3_, G_2_T_4_, and G_2_T_5_ displayed spectra characteristic of type III quadruplexes with strong positive peaks at 295 nm and negative peaks at 265 nm. These data are similar to that observed with G_3_-tract sequences [42]. We also found that sequences with at least one loop of three thymidines and one or two loops of two thymidines displayed spectra similar to that seen with G_2_T_3_, demonstrating that only one loop of three nucleotides is necessary for folding into a stable type III structure (Figure S1A). This suggests libraries with a minimum of three random nucleotides in one of their loops would be useful in an in vitro selection experiment as they form stable structures and do not form polymorphic mixtures of different quadruplex topologies. For the upper limit of loop length, we found that G_2_-tracts with loops of more than five thymidines showed a change in CD spectra suggesting these sequences are not forming quadruplex structures (Figure S1B). Studies on G_3_-tract sequences with loops longer than seven nucleotides show that they typically do not support quadruplex formation [40], [42]. To test for the maximum loop length that would support quadruplexes in a G_2_-tract library, we chose to test libraries with up to seven random nucleotides in their loop regions, as interactions between the random bases could potentially stabilize quadruplex structures.

The choice of loop lengths between three and seven residues also makes sense from the perspective of in vitro selection. First, the majority of efficient aptamers fall within this size range. Also, using these libraries will allow for the complete sampling of available sequence space, which would not be possible using larger libraries. Finally, as libraries increase in size and the random sequences represent higher proportions of the sequences compared to the fixed guanines, they will likely become more involved in the structural folding and the two-tiered quadruplex arrangement may be lost in favor of other structural arrangements.

### Determining Folding of G_2_-tract Libraries with Circular Dichroism

To examine the folding that would be present in a library containing four pairs of guanine residues we performed CD analysis on libraries containing three random regions flanked by four pairs of guanines (GGN_X_GGN_X_GGN_X_GG), represented as G_2_N_x_. CD is an appropriate technique in this endeavor as the concentrations necessary to obtain a readable spectrum (µM range) are similar to those used in the first round of selection where it is desirable to screen as many sequences as possible. The CD spectra of the G_2_N_3_ to G_2_N_7_ are shown in Figure 2B. Each scan contains a bimodal spectrum with positive ellipticity peaks at 264 and 295 nm of differing intensities. This could be explained by two scenarios. One scenario is that the majority of sequences are folded into type II quadruplexes, which would explain the positive ellipticity at 264 and 295 nm. In the second scenario a mixture of type I, II and III quadruplexes could be present, with their cumulative spectra averaging to create the positive peaks at 264 and 295 nm. This has been observed previously with sequences known to fold into heterogenous mixtures of different quadruplex structures and can be observed from our analysis of G_2_T_2_ (Figure 2A). This second scenario would also explain the differences in 264/295 nm ratios seen when comparing the spectra of the different loop libraries, as these libraries could contain different amounts of each type of quadruplex. For instance, the spectrum of G_2_N_3_ has a peak in ellipticity at 264 nm about twice the intensity of a second peak seen at 295 nm. When one random base is added to each loop to create the G_2_N_4_ library the spectrum is reversed with a peak in ellipticity at 295 nm that is twice as intense as the peak at 264 nm. It could be reasoned that this shift in spectra could be due to more sequences in the G_2_N_4_ library being folding into type III quadruplexes and less being folded into type I quadruplexes. This would explain the lowering of 264 nm ellipticity in G_2_N_4_ as less type I quadruplexes would be present to contribute to positive ellipticity at 264 nm and the increased number of type III quadruplexes would provide negative ellipticity lowering the ellipticity at 264 nm. To address which of the two scenarios is causing this effect, the folding of the individual sequences in the libraries will be addressed by analysis of a small library later in the manuscript.

In contrast to the G_2_-tract libraries, a random library of twenty-three nucleotides showed a spectrum with a single positive peak around 280 nm and no peaks at 264 and 295 nm. This suggests that the majority of sequences in this library are folded into other non-quadruplex structures. The lack of other peaks in the G_2_-tract libraries, such as the 280 nm peak seen with the completely random library, shows that there are not enough sequences folded into other structures to appear in the CD spectra of the G_2_-tract libraries suggesting these G_2_-tract libraries are enriched in quadruplex structures.

### Thermal Stability Test of G_2_-tract Libraries

**Figure 3:**
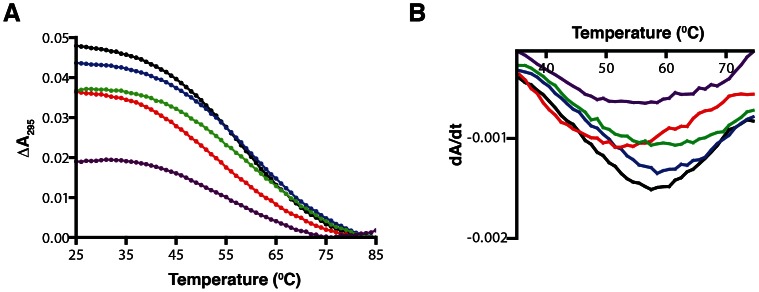
Melting profiles of G_2_-tract libraries. (A) The thermal stability of five G_2_N_X_ sequences in which X = 3, 4, 5, 6 and 7 were assessed by thermal denaturation analysis. The absorbance of each library was measured at 295 nm between 15 and 85°C. The difference in absorbance was measured as the difference between the highest and lowest A_295_ measurements for each sample. The color scheme is the same as in Figure 2. (B) The first derivative of the melting profiles was taken for each library. The most negative derivative value for each library represents the T_m_.

We next set out to assess the stability of the folded sequences within the libraries. It has been shown that quadruplexes absorb 50 to 80% more light at 295 nm compared with their unfolded counterparts [50] and A_295_ melting and annealing profiles have been used to train quadruplex stability algorithms [39]. Observing the change in absorbance at 295 nm during heating and cooling can assess the stability of a quadruplex. The absorbance of each G_2_-tract library was recorded between 15 and 85**°**C. As shown in Figure 3A, each library exhibited a decrease in absorbance as the temperature increased. This is consistent with the molecules within the libraries being folded into quadruplex structures. The melting profile of each library is broader than that typically observed with a single DNA sequence. This phenomenon has been observed previously with mixtures of different quadruplex sequences and suggests multiple structural species being present with similar melting temperatures [51]. The melting temperature (T_m_) of each library was determined by taking the minimum of the first derivative of the melting profile (Figure 3B). The melting temperatures for all G_2_-tract libraries with loops from three to seven nucleotides were all found to be between 50 and 60**°**C. This demonstrates that these libraries are comprised of quadruplex scaffolds at temperatures typically used for in vitro selection (between 16 and 37**°**C). We were also interested in determining whether the sequences in each library were folded into unimolecular quadruplexes or higher order structures such as bimolecular or tetramolecular quadruplexes. As shown in Figure S2, the melting and annealing profiles of each library showed some degree of hysteresis, which can be representative of unimolecular or multistranded structures [52]. To assess whether the libraries were folding into unimolecular or multistranded structures, melting profiles were collected at different oligonucleotide concentrations for each of the libraries. This can assess the molecularity of the quadruplexes present, as multistranded quadruplexes are more stable at higher oligonucleotide concentrations making their melting temperatures concentration dependant, while intramolecular interactions are not effected by oligonucleotide concentration, meaning unimolecular quadruplexes melting temperatures are not affected by concentration [52]. As seen in Figure S3, the melting temperature did not change when the library concentrations were doubled and quadrupled to 8 and 16 µM for the G_2_N_3_, G_2_N_4_, G_2_N_5_, and G_2_N_6_ libraries. This indicates that these libraries are comprised predominantly of quadruplexes folded into unimolecular quadruplex structures. For the G_2_N_7_ library, the melting temperature was found to increase by one degree each time the library concentration was doubled. This suggests that this library is folding into multistranded structures, likely due to the larger loops having more potential to form stable interactions with the loops of other strands. The dominance of unimolecular quadruplexes in the G_2_N_3_ to G_2_N_6_ libraries shows that these libraries would be useful for in vitro selection as most selection schemes are designed to select for unimolecular functional nucleic acids.

### Examining the Folding of a Collection of Randomly Generated Sequences

**Figure 4:**
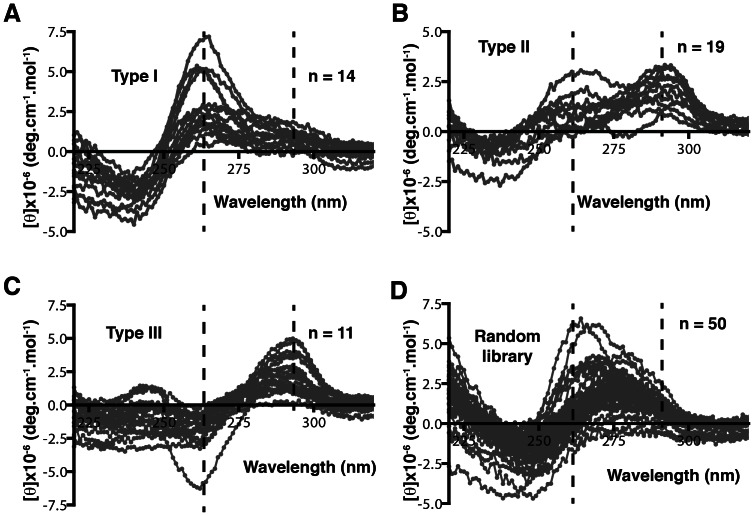
Analysis of 50 random sequences with and without G_2_-tracts. Circular dichroism analysis was carried out on 50 randomly generated sequences based on G_2_N_5_ (A-C) or N_23_ (D). From the G_2_N_5_ collection, 14, 11 and 19 sequences (44 in total) may adopt a type I, II or III quadruplex structure, based on the observation that their spectra produced a positive peak near 264 nm (A), two positive peaks at 264 and 295 nm (B), or a positive peak at 295 nm and a negative peak at 265 nm (C). In contrast, only 6 out of the 50 sequences from the N_23_ collection may adopt a quadruplex structure (a positive peak around 264 nm), while the remaining 44 sequences did not appear to form a quadruplex structure as these sequences produced a peak at 280 nm but no peaks at 264 and 295 nm (D).

The work described so far has been performed on a mixture of a library of 10^13^ sequences with the data presented representing an accumulation of the properties of each sequence within the library. To investigate the behaviour of individual sequences in a library, we analysed 50 G_2_-tract sequences with randomly generated loop regions by CD. The sequences were designed based on the G_2_N_5_ library, containing three random regions of five nucleotides each. This library was chosen for further analysis as its CD spectrum indicated that it showed nearly equal amounts of ellipticity at 264 and 295 nm suggesting it may contain a mixture of molecules folded into different types of quadruplexes. By analysing individual sequences, it can be determined whether this represents a mixture of type I, type II and type III arrangements or a homogenous population of type II quadruplexes that would also explain this spectrum. The 50 sequences were generated using rsat [53] (Table S1) and each was individually scanned by CD between 320 and 220 nm. 44 of the 50 sequences gave a profile that was indicative of a quadruplex structure. Each sequence was classified by the its CD profile, with sequences with a 264 nm maximum classified as type I quadruplexes, sequences with positive ellipticities at 264 and 295 nm being classified as type II quadruplexes, and sequences with positive ellipticitity at 295 nm and negative ellipticity at 264 nm being classified as type III quadruplexes. As seen in Figure 4, 14 of the sequences adopted a type I conformation (Figure 4A), 19 folded into a type II arrangement (Figure 4B), and 11 sequences showed spectra indicative of a type III conformation (Figure 4C). The presence of all three of the possible quadruplex structural topologies in roughly equal amounts suggest that this library has the variety of arrangements necessary for it to be useful for an in vitro selection. In contrast, in a screen of 50 completely random sequences of the same length (Table S2), only 6 sequences displayed a peak maximum near 264 nm indicative of a type I quadruplex, while the other 44 sequences had a maximum around 280 nm. This suggests that the majority of randomly generated sequences are not folding into quadruplex structures (Figure 4D). For comparison with the library scans in Figure 2B, the spectra of the 50 G_2_-tract sequences were summed and divided by the total number of sequences. As shown in Figure S4, the profile contains a peak at 295 nm with a shoulder at 265 nm similar to that observed with the random sequence libraries. When the spectra of the 50 completely random sequences were combined they yielded a profile with a peak around 280 nm, also similar to their random library counterpart. The observation that both the combined spectra of the 50 G_2_-tract sequences and of the 50 random sequences resembled the spectra generated with their respective libraries validates the utility of CD scans of libraries as an indicator of the percentages of different types of quadruplex structures present within each pool of sequences.

### Assessing the Utility of G_2_-Tract Libraries for in vitro Selection

**Figure 5:**
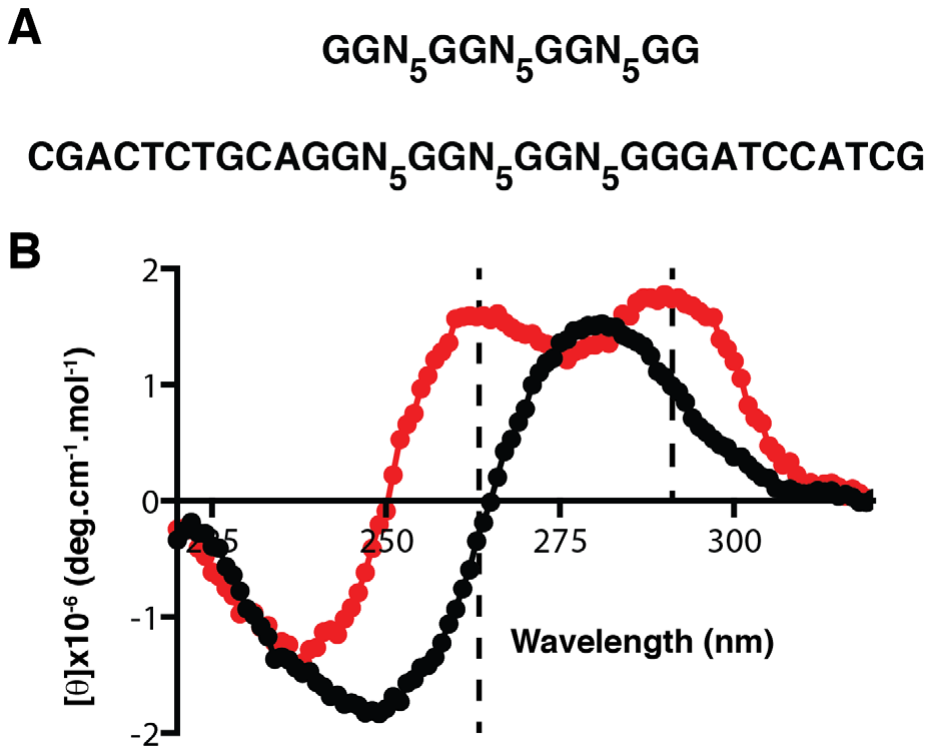
Effects of primer binding sites on G_2_-tract library folding. (A) Sequences of the G_2_N_5_ library and a library with fixed 5′ and 3′ regions. (B) CD analysis of G_2_N_5_ (red) and G_2_N_5_ with the two fixed sequences (black).

**Figure 6:**
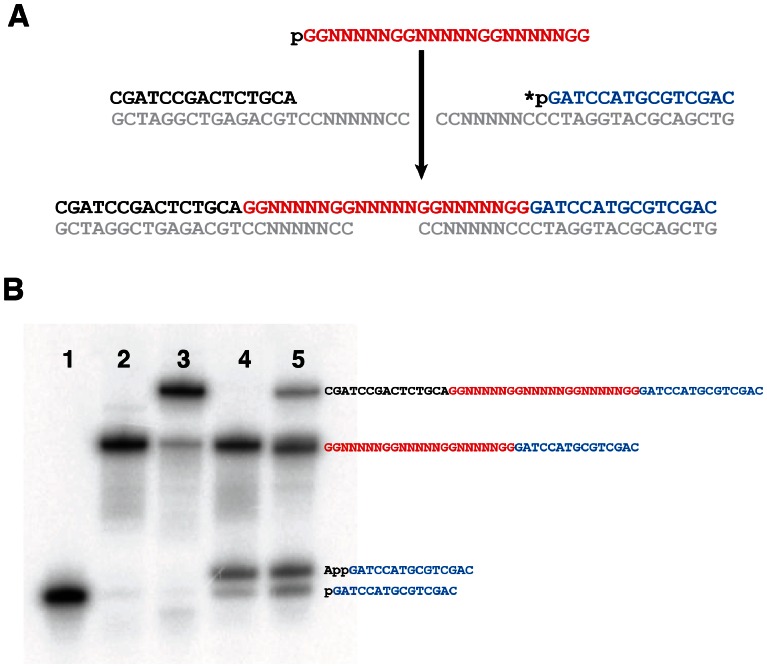
Scheme for the ligation of the representative sequence DGR36 to PCR adapters. (A) DGR36 (red) was ligated to 5′ (black) and 3′ (blue) adapters, with two template oligonucleotides (grey). The asterisk represents a ^32^P labelled phosphate. (B) Ligation of one and two adapters to DGR36. The 3′ adapter was labelled with ^32^P (lane 1) and incubated with T4 DNA ligase, DGR36 and fully complementary template (lane 2), as well as all these oligonucleotides plus a 5′ adapter and fully complementary template (lane 3). The same reactions were performed with the fully complementary templates replaced with partially randomized 3′ templates (lane 4) and both 3′ and 5′ templates (lane 5). The p and App on the 3′ adapters in lanes 4 and 5 represent 5′ phosphorylated and 5′ adenylated adapters, respectively.

While CD and UV melting analyses suggest that libraries containing four G_2_-tracts are comprised primarily of DNA molecules folded into quadruplex structures, the libraries need to possess other attributes to be useful for in vitro selection. First and foremost, as in vitro selection contains multiple rounds of partition and amplification, sequences from the library must be capable of being amplified after each partition step to allow for the enrichment of active sequences. This is typically achieved by the addition of fixed sequences to the 5′ and 3′ ends of the library. These sequences serve as primer-binding sites, allowing for the amplification of active sequences by PCR. We first tested whether addition of fixed sequences at the 5′ and 3′ ends affected the folding of the G_2_-tract libraries. As shown in Figure 5A, 10 nucleotides were added to the 5′ and 3′ of the G_2_N_5_ library. CD spectrometry was performed on this library and compared to the original G_2_N_5_ library, which lacked primer-binding arms. As seen in Figure 5B, when the fixed arms were added the spectrum shifts from the mixed quadruplex profile to a profile similar to the completely random library. This suggests that the majority of sequences within the library are forming interactions between the fixed sequence domains and the random nucleotides. If this library were to be used for in vitro selection, many of the quadruplex scaffolds that were present in the library without the fixed sequences would not form and be lost during the partition step. As an alternative, we considered the possibility of ligating primer-binding sequences to the 5′ and 3′ ends of sequences within the G_2_N_5_ library. This would allow the primer binding sites to be added after the partition step and prevent them from disrupting the folding of sequences into quadruplex structures during the selection steps. The ligation scheme is shown in Figure 6A. The specific ligation of the primer-binding sites to sequences in the G_2_N_5_ library is achieved using two DNA oligonucleotides containing regions complementary to the primer-binding sites along with two dicytosines flanking a five nucleotide random region as templates. While performing template-directed ligation with randomized regions might seem problematic, in this case the random regions only need to hybridize to five base pairs. Therefore there are only 1024 possible complementary sequences, meaning there are 6×10^11^ complementary sequences present in a one picomole reaction. The specificity of the templates for the library is also conferred by the terminal dicytosine in the template, which can bind to the central G_2_-tracts within the library. To test whether ligation of a sequence was possible using partially randomized templates, the ligation of a sequence from the randomly generated library was incubated with 5′ and 3′ adapter sequences and two partially randomized templates as shown in Figure 6A. The sequence (DGR36, Table S1) was selected as its CD spectrum suggested that it folded into a type III quadruplex similar to those structures seen with known quadruplex aptamers. The results of the ligation are shown in Figure 6B. Well-defined products were seen when one or two oligonucleotides were ligated to the library, showing that specific ligation can be carried out with a randomized region. The efficiency of the ligation was lower than that achieved when the completely complementary template was used, as seen in lanes 3 and 5 of the figure. As the library contains only 10^9^ sequences, one nanomole of library will contain 10^6^ copies of each individual sequence, so this lower efficiency of ligation should still allow for the amplification of all unique sequences that survive the partition step of the first round of a selection. The presence of a ligation product also shows that this sequence with a strong tertiary quadruplex structure can successfully hybridize to the template in an orientation that allows for ligation.

### Amplification and Regeneration of G_2_-Tract Libraries

**Figure 7:**
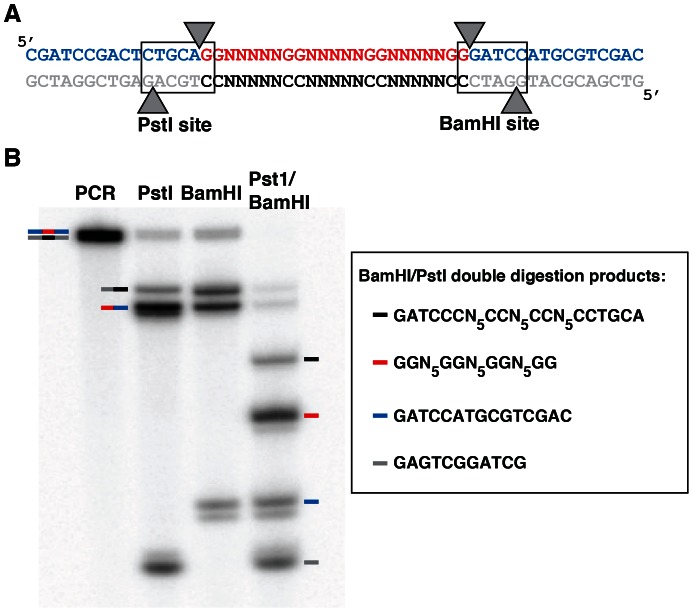
Regeneration of G_2_N_5_ population from PCR products. (A) Sequence of the PCR product. PstI and BamHI restriction sites are shown in boxes. Restriction enzyme cleavage sites are represented by arrows. (B) Digestion of amplified DGR36 sequence with BamHI and PstI individually (lanes 2 and 3) and in combination (lane 4). Coloured arrows indicate fragments comprised of sequences of the same colour in Panel A. Fragments consisting of only the first fifteen nucleotides of the 5′ regions of the PCR products in the single enzyme digestion experiments cannot be seen as only polymerized segments acquire ^32^P during amplification.

The ligated DNA molecules were amplified by PCR, using two primers that hybridize to the ligated primer-binding sites of the sense and antisense strand of the G_2_-tract sequence (Figure 7A). For successive rounds of selection to be undertaken, it is crucial that the segment corresponding to the initial population be separated from its complementary strand. Moreover, in the case of a G_2_-tract population, the fixed regions at the 5′ and 3′ ends must be removed prior to the next selection step, as they will interfere with the folding of the active sequences. In typical aptamer selections, the library strand is separated from its antisense strand by methods such as affinity chromatography of a biotin labeled antisense primer. Methods exist for removing a 5′ sequence from an amplified library, such as incorporation of a ribonucleotide into the reverse PCR primer that can be cleaved by sodium hydroxide treatment [28]. Removing the 3′ fixed region, which is at the end of the amplified product, is typically not performed and requires a novel method for removal.

To accomplish the removal of both the 5′ and 3′ fixed ends as well as the separation of the library strand from its antisense counterpart after PCR, restriction sites were incorporated into the sequences as shown in Figure 7A. The 5′ primer contains a PstI site (CTGCAΔG) which encompasses the 5′ terminal guanine of the library and five nucleotides of the fixed 5′ region; the 3′ primer consists of a BamHI site (GΔGATCC), which contains the 3′ terminal guanine and five nucleotides of the 3′ fixed region. Digestion with PstI and BamHI following PCR amplification leads to cleavage of the product into several fragments. Since the restriction enzymes cut each strand at different sites, the 23-nucleotide library can be separated from the antisense strand by denaturing PAGE as the library will be seven nucleotides shorter. The band distribution observed following PstI and BamHI digestion of the amplified DGR36 sequence is shown in Figure 7B. When the PCR product was digested with PstI or BamHI individually, the bands corresponding to the fragments of a single digestion were seen. When the PCR product was digested with both restriction enzymes, a band corresponding to the library was seen. This resulting population of amplified sequences from the G_2_N_5_ library can then be used to initiate the next round of selection.

## Discussion

*In vitro* selection has established itself as a powerful technique for the isolation of a wide range of functional nucleic acids. Existing aptamers isolated by in vitro selection have been coupled to a number of signal transducing strategies, producing sensors that have the potential to be used in many practical applications. However, despite this progress, there has been difficulty in isolating aptamers for certain targets or with a desired affinity and specificity. If these obstacles can be overcome and aptamers with the desired properties can be obtained for any target, the future potential of this class of binders could be limitless. By understanding which structural arrangements are effectively used by aptamers, it may be possible to modify in vitro selection protocols to favor these types of arrangements. One type of structure, the guanine quadruplex has been seen repeatedly in the core of a number of functional DNA molecules. By analyzing DNA oligonucleotide libraries with four G_2_ sequence elements we were able to demonstrate that the majority of molecules fold into quadruplex structures that are stable at temperatures typically used for in vitro selection. The libraries were also found to have features of all three types of known quadruplexes, suggesting a mixture of these structures in the libraries. Analysis of 50 representative sequences from the G_2_N_5_ library revealed that roughly equal number of sequences folded in type I, type II, and type III quadruplexes. This diversity of quadruplex scaffolds translates to a diversity of loop arrangements in quadruplexes within the libraries. In an in vitro selection experiment, these loop residues represent potential binding partners with the target ligand. The more loop arrangements and combinations of loop arrangements present within molecules within the library, the greater the chance of a sequence being present in the library that can bind a target ligand. We have also devised a scheme for ligation of primer-binding sites after a partition step to avoid fixed sequence elements being present during selection, as we found these sequences to be detrimental to quadruplex formation. Providing libraries with a structural scaffold shall reduce the burden of potential aptamers to use their random residues for intramolecular folding and allow the random bases to be used solely for target binding, which in turn shall allow for the selection of highly selective, high-affinity aptamers.

## Materials and Methods

### Oligonucleotides and Materials

DNA oligonucleotides were prepared using standard phosphoramidite chemistry (MOBIX Lab, McMaster University, Hamilton, ON, Canada; and Integrated DNA Technologies, Coralville, IA, USA). The partially randomized G_2_-tract libraries were synthesized using a 25% mixture of each of the four nucleotides at the random positions. Each oligonucleotide was purified by 10% denaturing (8 M urea) polyacrylamide gel electrophoresis (PAGE), and its concentration was determined spectroscopically. T4 polynucleotide kinase (PNK) and T4 DNA ligase were purchased from MBI Fermentas (Burlington, ON, Canada). Taq polymerase, BamHI and PstI were purchased from New England Biolabs (Pickering, ON, Canada). [γ-^32^P]ATP and [α-^32^P]deoxy-GTP were purchased from Perkin Elmer (Woodbridge, ON, Canada). All other chemicals were obtained from Sigma-Aldrich (Oakville, ON, Canada).

### Buffer Conditions

For the CD and UV melting experiments, the analyses were performed under the following conditions: 500 mM KCl, 10 mM MgCl_2_, and 25 mM HEPES, pH 7.

### CD Analysis

CD studies were carried out using an AVIV model 410 CD spectrometer (Lakewood, NJ, USA) as per the manufacturer’s instructions. All constructs were scanned from 320 to 220 nm at 10 µM DNA concentration in a 0.1 cm quartz cuvette. All DNA samples were heated to 90°C in 1× buffer and allowed to cool to room temperature. All shown spectra are the average of three individual scans.

### UV Melting Profiles

Thermal denaturation profiles were carried out using a Cary 100-bio UV spectrometer (Mississauga, ON, Canada) as per the manufacturer’s instructions. All libraries were incubated in 1× buffer at concentrations of 4 µM, 8 µM and 16 µM. Oligonucleotides were heated and cooled at a rate of 0.5°C per min and the absorbance was measured at 1°C intervals. First derivative curves were calculated using Graphpad Prism software.

### DNA Phosphorylation, Ligation, Polymerase Chain Reaction and Restriction Digestion

Oligonucleotides for the ligation of DGR36 to 5′ and 3′ PCR adapters are shown in Figure S5A. Before ligation, 50 pmol of DGR36 and 3′ adapter sequence were phosphorylated at their 5′ ends with PNK. The sequences were heated to 90°C and cooled to room temperature before PNK treatment. DGR36 was incubated with 10× PNK buffer A (MBI Fermentas), 1 mM ATP and 10 U of PNK at 37°C for 30 min. The reaction was then heated at 90°C for 5 min to deactivate the PNK. The 3′ adapter was treated in the same way except 1 µL of [γ-^32^P]ATP instead of 1 mM ATP was used to allow for radiolabeling of the adapter. Ligations were carried out using 50 pmol of each oligonucleotide shown in Figure S5A. Oligonucleotides were heated to 90°C for 1 min and cooled to room temperature. 10× T4 DNA ligase buffer and 1 U of T4 DNA ligase were added and the reaction was incubated for 1 h at room temperature. Each reaction mixture was precipitated with ethanol and the DNA was resuspended in 10 µL of 1× loading buffer and resolved by 10% denaturing PAGE. Ligated products were extracted from the gel for subsequent PCR amplification. PCR primers used for amplification of ligated G_2_N_5_ library are shown in Figure S5B. 50 pmol of forward and reverse primers were mixed with a 1000× dilution of the ligated product, 200 µM dNTPs, 1 µL of [α-^32^P]deoxy-GTP, 10× Taq DNA polymerase buffer with MgCl_2_ and 1 U of Taq DNA polymerase. Twenty cycles of PCR were performed. The DNA in the reaction mixture was precipitated with ethanol and digested using 20 U of BamHI and PstI in NEB Buffer 3 supplemented with BSA.

## Supporting Information

Figure S1 (A) Circular dichroism (CD) analysis of G_2_-tract (GGT_X_GGT_X_GGT_X_GG) with X = 2 or 3. CD of sequence with two thymidines in each loop region (G_2_T_2_) is shown in red. Scans of all other combinations of two or three thymidines in each loop are shown in black. Dotted lines are placed at 264 and 295 nm as ellipticities at these wavelengths indicate the presence of different topologies of quadruplexes. (B) CD analysis of G_2_-tract sequences with longer thymidine loops. G_2_T_5_ (red) shows a spectrum characteristic of a type III quadrupulex, while sequences with longer thymidine loops, G_2_T_6_ (green), G_2_T_7_ (purple), and G_2_T_8_ (black) show spectra not suggestive of quadruplex formation.
(TIF)

Figure S2 **Annealing and melting curves of G_2_N_3_ to G_2_N_7_ recorded at 295 nm.** Black lines indicate melting curves and red lines indicate annealing curves. (A) G_2_N_3_, (B) G_2_N_4_, (C) G_2_N_5_, (D) G_2_N_6_, and (E) G_2_N_7_.
(TIF)

Figure S3 **Melting profiles were obtained for G_2_N_3_ to G_2_N_7_ at 295 nM at DNA concentrations of 4 uM (red), 8 uM blue, 16 uM (black). (A) G_2_N_3_, (B) G_2_N_4_, (C) G_2_N_5_, (D) G_2_N_6_, and (E) G_2_N_7_.**
(TIF)

Figure S4 **Average CD of 50 randomly generated G_2_-tract sequences.** The CD spectra of 50 randomly generated diguanine repeat sequences based on G_2_N_5_ were summed and divided by 50 to generate an average spectrum shown in red. 50 randomly generated 23 nucleotide sequences were scanned by CD and averaged for comparison and shown in black.
(TIF)

Figure S5 **Oligonucleotides used for ligation and PCR of DGR36.** (A) Oligonucleotides used for ligation of DGR36 to 5′ and 3′ adapters (5PA and 3PA). Oligos 5TM and 3TM are used as templates. (B) Oligonucleotides used for PCR of ligated G_2_N_5_ library.
(TIF)

Table S1 **50 Randomly generated G_2_-tract sequences based on GGN_5_GGN_5_GGN_5_GG used for CD analysis.** Fixed G_2_ motifs are shown in red.
(DOCX)

Table S2 **50 randomly generated 23 nucleotide sequences used for CD analysis control sequences.**
(DOCX)
