# Supplementary figures and images for: Assessing the Amount of Quadruplex Structures Present within G2-Tract Synthetic Random-Sequence DNA Libraries
Source: PLoS One. 2013 May 24;8(5):e64131. doi: 10.1371/journal.pone.0064131 (PMC3663748; doi:10.1371/journal.pone.0064131)

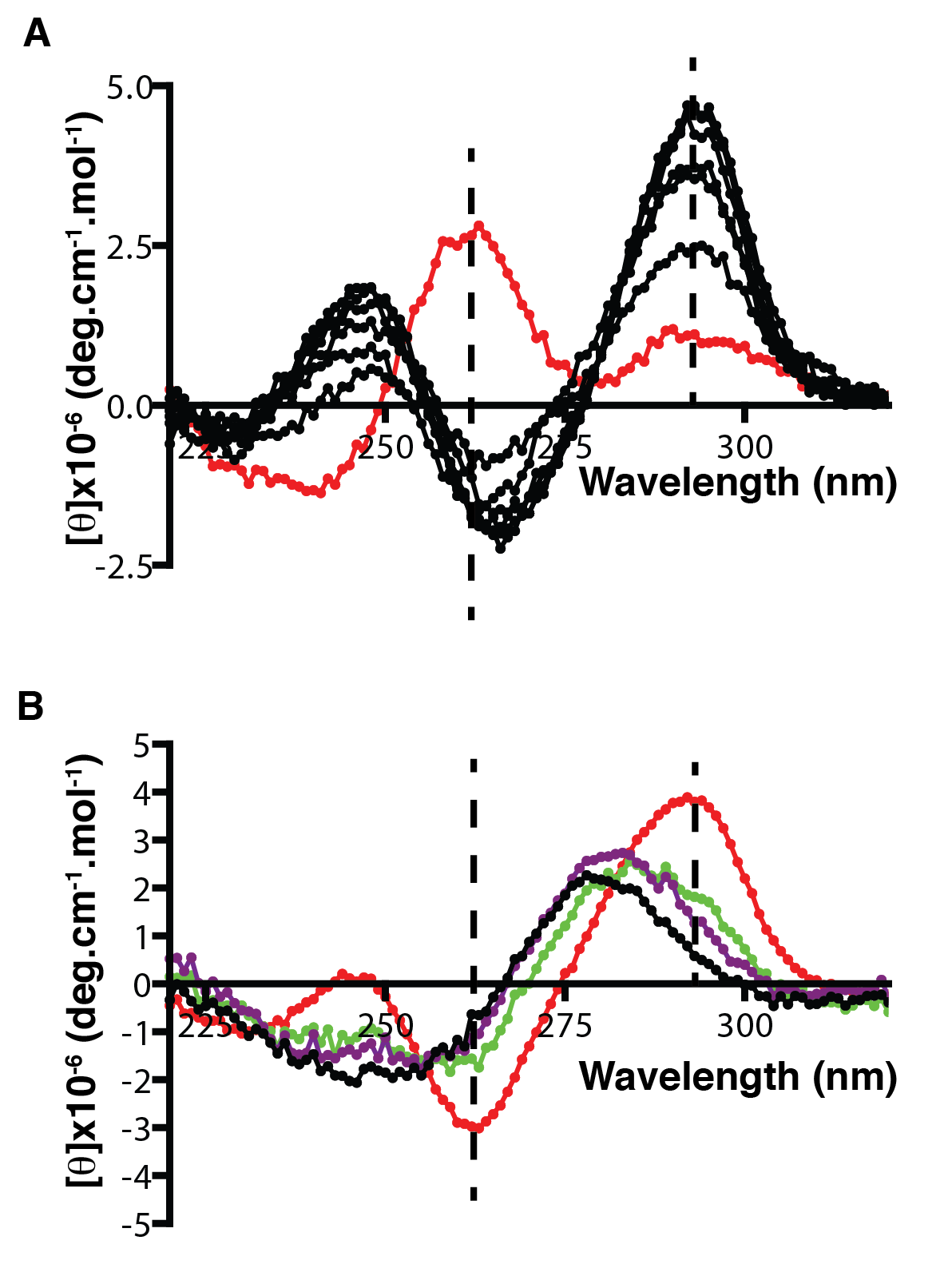

Supplement: Figure S1 — (A) Circular dichroism (CD) analysis of G2-tract (GGTXGGTXGGTXGG) with X = 2 or 3. CD of sequence with two thymidines in each loop region (G2T2) is shown in red. Scans of all other combinations of two or three thymidines in each loop are shown in black. Dotted lines are placed at 264 and 295 nm as ellipticities at these wavelengths indicate the presence of different topologies of quadruplexes. (B) CD analysis of G2-tract sequences with longer thymidine loops. G2T5 (red) shows a spectrum characteristic of a type III quadrupulex, while sequences with longer thymidine loops, G2T6 (green), G2T7 (purple), and G2T8 (black) show spectra not suggestive of quadruplex formation. (TIF) [file pone.0064131.s001.tif]

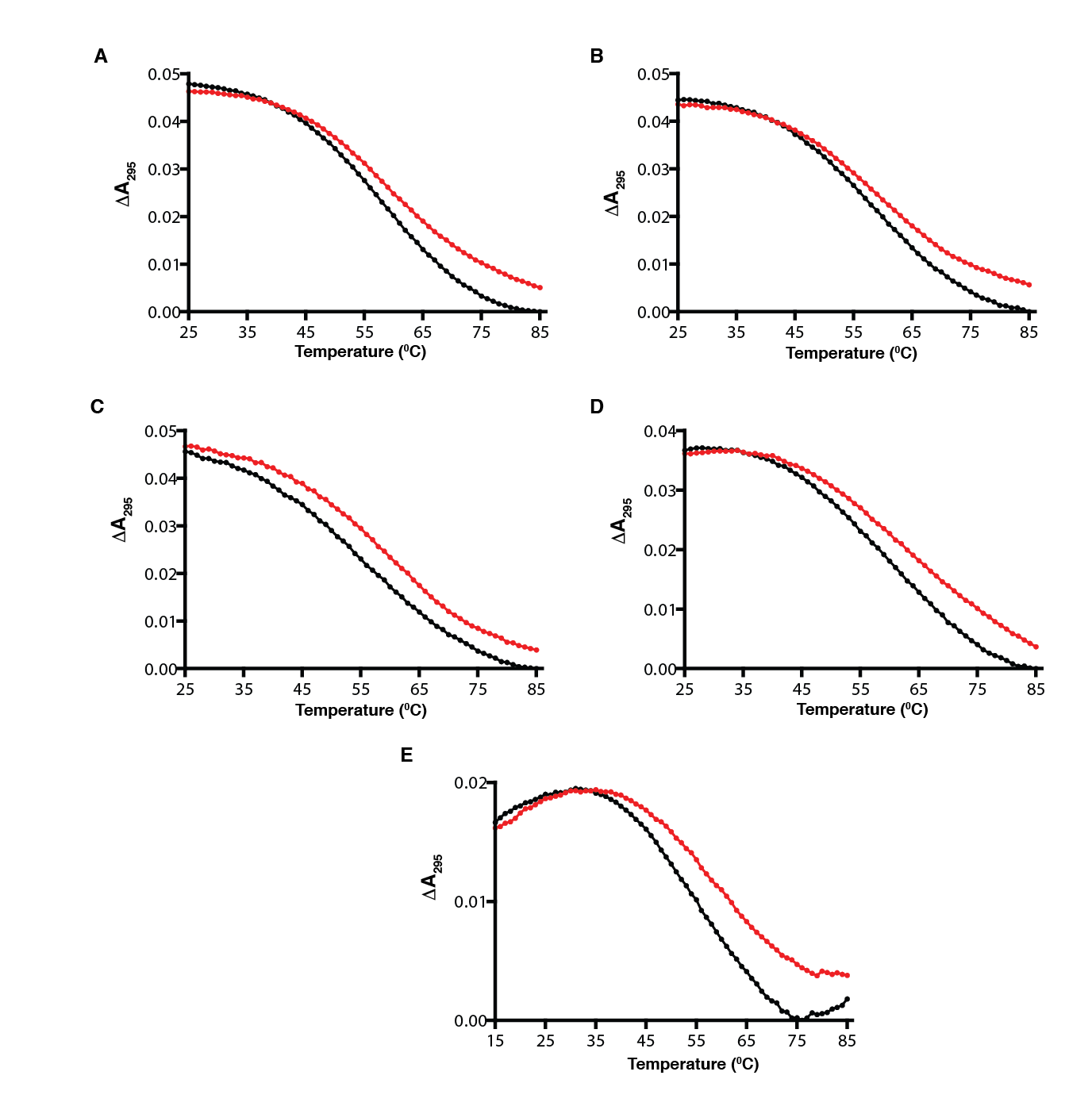

Supplement: Figure S2 — Annealing and melting curves of G2N3 to G2N7 recorded at 295 nm. Black lines indicate melting curves and red lines indicate annealing curves. (A) G2N3, (B) G2N4, (C) G2N5, (D) G2N6, and (E) G2N7. (TIF) [file pone.0064131.s002.tif]

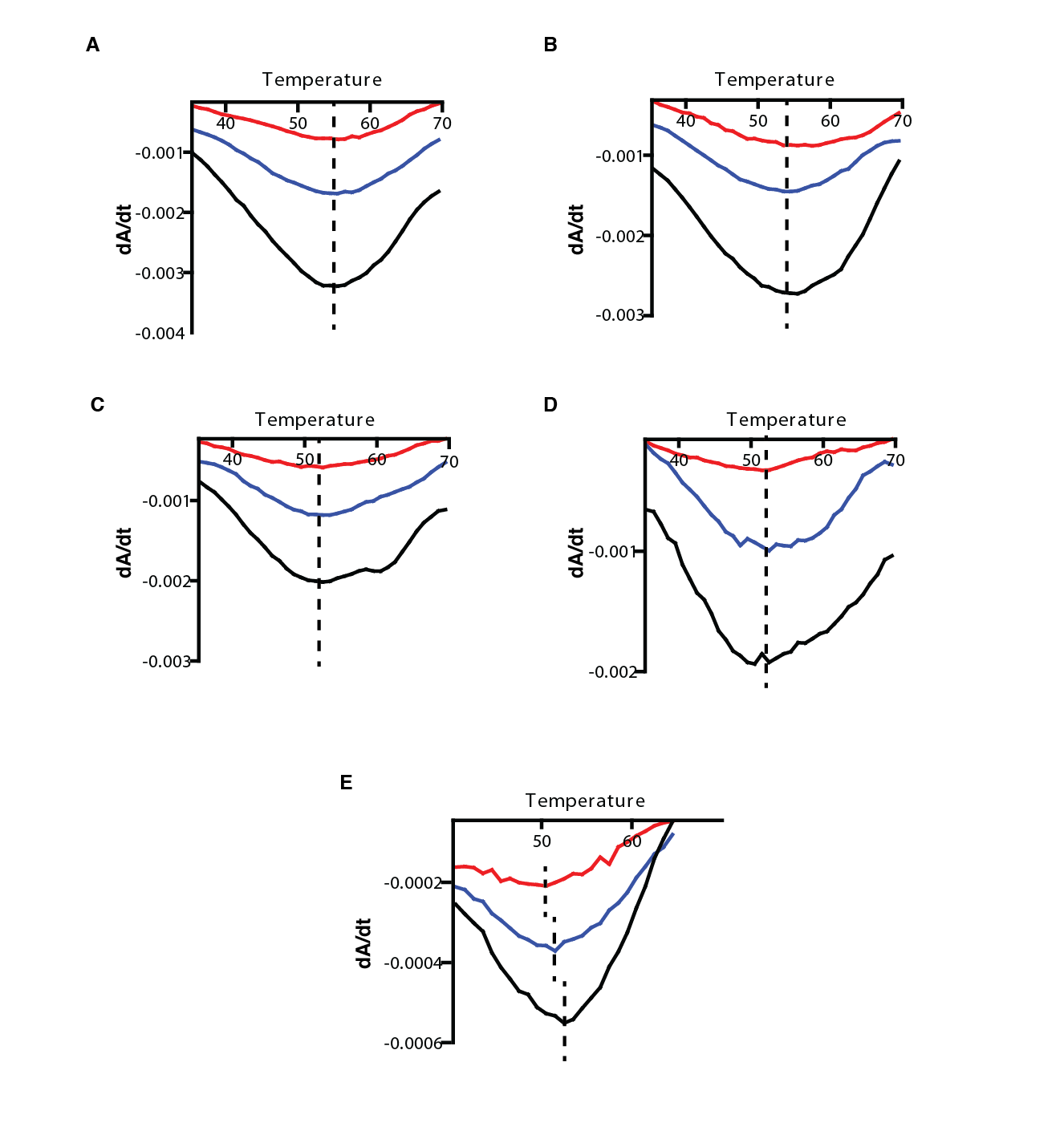

Supplement: Figure S3 — Melting profiles were obtained for G2N3 to G2N7 at 295 nM at DNA concentrations of 4 uM (red), 8 uM blue, 16 uM (black). (A) G2N3, (B) G2N4, (C) G2N5, (D) G2N6, and (E) G2N7. (TIF) [file pone.0064131.s003.tif]

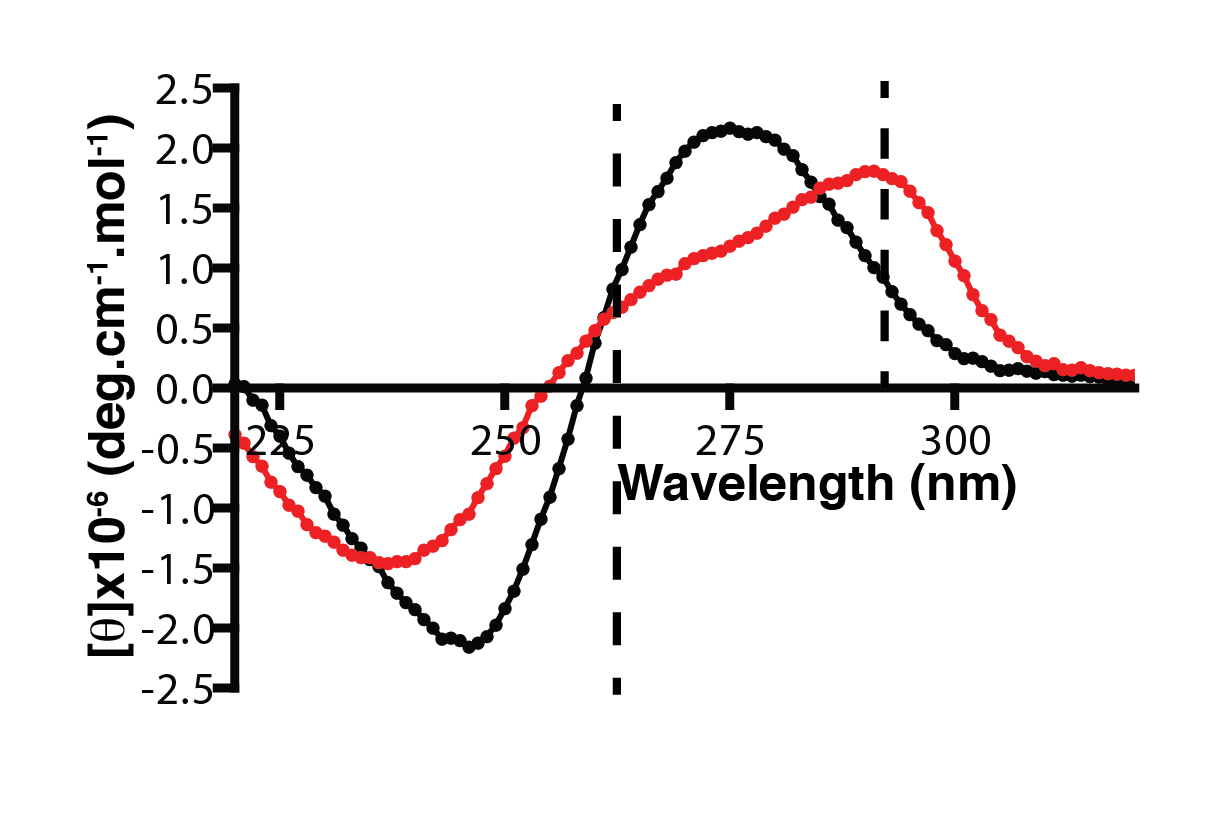

Supplement: Figure S4 — Average CD of 50 randomly generated G2-tract sequences. The CD spectra of 50 randomly generated diguanine repeat sequences based on G2N5 were summed and divided by 50 to generate an average spectrum shown in red. 50 randomly generated 23 nucleotide sequences were scanned by CD and averaged for comparison and shown in black. (TIF) [file pone.0064131.s004.tif]

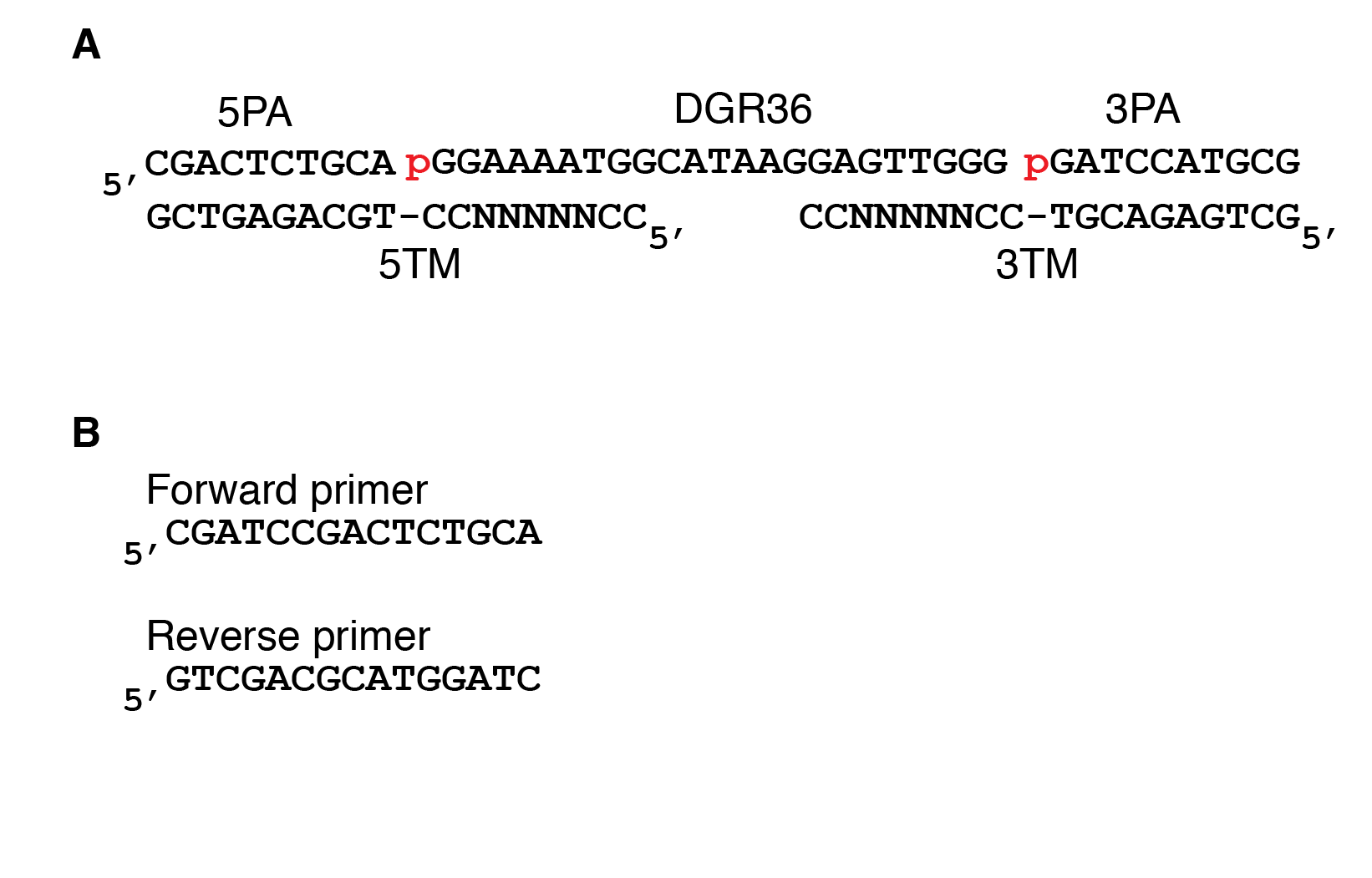

Supplement: Figure S5 — Oligonucleotides used for ligation and PCR of DGR36. (A) Oligonucleotides used for ligation of DGR36 to 5′ and 3′ adapters (5PA and 3PA). Oligos 5TM and 3TM are used as templates. (B) Oligonucleotides used for PCR of ligated G2N5 library. (TIF) [file pone.0064131.s005.tif]
