# Supplementary material for: Assessing the Amount of Quadruplex Structures Present within G2-Tract Synthetic Random-Sequence DNA Libraries
Source: PLoS One. 2013 May 24;8(5):e64131. doi: 10.1371/journal.pone.0064131 (PMC3663748; doi:10.1371/journal.pone.0064131)
Supplement: Table S1 — 50 Randomly generated G2-tract sequences based on GGN5GGN5GGN5GG used for CD analysis. Fixed G2 motifs are shown in red. (DOCX) [file pone.0064131.s006.docx]

| Name | Sequence | Name | Sequence |
| --- | --- | --- | --- |
| DGR1 | GGCAATAGGCGTAAGGTCTACGG | DGR26 | GGGTAGGGGGCCCTGGCAGGGGG |
| DGR2 | GGAATAGGGGGGTCGGTGCCGGG | DGR27 | GGATAACGGCGTTAGGAATCGGG |
| DGR3 | GGCTCCAGGTTCGCGGATCACGG | DGR28 | GGTAGACGGTCCTCGGTATGAGG |
| DGR4 | GGCGAAGGGGAACTGGGGTGGGG | DGR29 | GGTAAAAGGCAAATGGATACTGG |
| DGR5 | GGGTTATGGACTGGGGTTCGAGG | DGR30 | GGACGTAGGCTGTGGGCCTTTGG |
| DGR6 | GGGGGCGGGGTCACGGAGTTGGG | DGR31 | GGATGACGGTGGCAGGCGTGCGG |
| DGR7 | GGAGATAGGGAACGGGTCACTGG | DGR32 | GGCATAAGGAGAAGGGGGTAAGG |
| DGR8 | GGGCGCGGGGAAACGGAAGTTGG | DGR33 | GGTAGTGGGCGCAAGGCTTCAGG |
| DGR9 | GGCTGACGGGACCGGGAAGAAGG | DGR34 | GGGGATGGGCACTCGGAACTAGG |
| DGR10 | GGATCTTGGGTCACGGTTGAGGG | DGR35 | GGCCGCCGGTTGCGGGGAATAGG |
| DGR11 | GGATACGGGCGTTGGGCGCGTGG | DGR36 | GGAAAATGGCATAAGGAGTTGGG |
| DGR12 | GGTATCTGGCCGGAGGGCCTAGG | DGR37 | GGTGAACGGTCCGTGGAATCCGG |
| DGR13 | GGGGATTGGAAGGCGGCCGGGGG | DGR38 | GGAATAGGGTGTGAGGGGACAGG |
| DGR14 | GGGTGTCGGACGCCGGTCTTGGG | DGR39 | GGGCAAAGGTAACGGGCCCCCGG |
| DGR15 | GGCACATGGGTTAAGGTCGAGGG | DGR40 | GGTGGCAGGGGAATGGCAGACGG |
| DGR16 | GGATGTCGGAGATTGGCCAGTGG | DGR41 | GGGGTCACGGTCTGTGGCCTCGG |
| DGR17 | GGGCCGCGGCACCGGGGCTTAGG | DGR42 | GGGCTCGGGGGATTGGGGTTAGG |
| DGR18 | GGCCGGAGGTTGTTGGGGTGTGG | DGR43 | GGGGCGAGGTCAGAGGCAAGGGG |
| DGR19 | GGAAAGGGGAACCCGGTGCGCGG | DGR44 | GGTTTAGGGCGCCCGGAGGCTGG |
| DGR20 | GGATGTAGGTGTCTGGTAAACGG | DGR45 | GGGAACCGGTGTGAGGTAGACGG |
| DGR21 | GGAAAGAGGCTGGCGGGAACCGG | DGR46 | GGCTTTAGGCGAACGGGCTCTGG |
| DGR22 | GGTCAGCGGTATGAGGTGTATGG | DGR47 | GGCCATGGGATTTAGGCCTGTGG |
| DGR23 | GGGGGACGGAATAAGGTTTGGGG | DGR48 | GGGATTCGGTAGCTGGCTCCTGG |
| DGR24 | GGTAAGAGGAAGATGGTTCGTGG | DGR49 | GGTTGGGGGAATACGGCCGAGGG |
| DGR25 | GGCACTTGGGGTGCGGGGAGGGG | DGR50 | GGCTCATGGTGTGCGGAATCCGG |
