# Supplementary material for: Assessing the Amount of Quadruplex Structures Present within G2-Tract Synthetic Random-Sequence DNA Libraries
Source: PLoS One. 2013 May 24;8(5):e64131. doi: 10.1371/journal.pone.0064131 (PMC3663748; doi:10.1371/journal.pone.0064131)
Supplement: Table S2 — 50 randomly generated 23 nucleotide sequences used for CD analysis control sequences. (DOCX) [file pone.0064131.s007.docx]

| Name | Sequence | Name | Sequence |
| --- | --- | --- | --- |
| RANDOM1 | GAATTGGCATGGGGTCTGACTCC | RANDOM26 | GCACAGGTTCGTGGATGTCTTGT |
| RANDOM2 | GCCCGGCGAAGAGCGGAGCAGCA | RANDOM27 | GGGGTGGTTACTCGGAGTAGTTT |
| RANDOM3 | TCCACGGCGGAAATATTTCATAA | RANDOM28 | TGCTGGTTCAGTCCGTAACAGAT |
| RANDOM4 | TCAAGTACCAGTTTAGCTGAGCA | RANDOM29 | AAATGGAAACCGAACCTAAATCC |
| RANDOM5 | TGAATAGCAGATCCGCCCTGGTC | RANDOM30 | CACCATAGTTATAGTTACCTGAT |
| RANDOM6 | CACCCTCACCCCCGAGTATTCGT | RANDOM31 | AGGCCCTAAAATGCGGTAGGCCT |
| RANDOM7 | GATAGTAAGTCCTAAACTTTCTG | RANDOM32 | AATGAACGACACTTAACGCCTAT |
| RANDOM8 | ACATGTCTATGATTATGTATGAA | RANDOM33 | TGGTAATCATCGACACGGGTCAA |
| RANDOM9 | TAGGATACAAATGCCGATAGCTC | RANDOM34 | AATTTGTCAGCGAAGCGTGCTCA |
| RANDOM10 | TAGGATACAAATGCCGATAGCTC | RANDOM35 | TGACCCATATCTATCTGTGTCCC |
| RANDOM11 | GATTGGATTAGTATGTCCCCTCT | RANDOM36 | ATACGGGGGTTATCTGGTCTGTC |
| RANDOM12 | TAAGTCTAGCGGGGTTGCACTCG | RANDOM37 | ACAGATTGCGATTAATGCTAACG |
| RANDOM13 | GGGAAGGAAACACCCGCACAGTC | RANDOM38 | CTCGCGGTTAAGCCACCCCGGGT |
| RANDOM14 | GGTTGCGGCGCAGAAACGAACTC | RANDOM39 | CCGGCACCCGGACCTGTTACGTC |
| RANDOM15 | AAATTCCTCGCTCCTGAACGTAC | RANDOM40 | ATCGGGTCACCGTCCACTGCTTG |
| RANDOM16 | GTATAAGGACAACAAAGCGTGCT | RANDOM41 | AGACGCTCTTGAGACTTCTAACA |
| RANDOM17 | CGGCTTGAAGTGAATTGCGTGAA | RANDOM42 | GGTATTGGGAGTCGTATATTACT |
| RANDOM18 | ATACCGGTTGCGCACACGGCATC | RANDOM43 | ATTGTCCTGACAAGATTCGGCTC |
| RANDOM19 | AGTCGCGGCCCGAGGGCTCGATC | RANDOM44 | TTGTACAGTGTCGTATGCACCTG |
| RANDOM20 | CTACGATTCACGCGGTCTAGCTT | RANDOM45 | TTGTGTTTCCCCTTGACCGGAGT |
| RANDOM21 | GCTCGCACGAGTGGTCTATTTTG | RANDOM46 | CGAGGCGACGCTCCTTTTACGCT |
| RANDOM22 | AAAGTCCTTGTTGACGGTAGAGC | RANDOM47 | GTGATGCGGCCGAAGAATGAAAC |
| RANDOM23 | CCGGGAAGAGAGGATGGGGCGCT | RANDOM48 | ACGCGATAACCGGTTGAGTGTCT |
| RANDOM24 | GTTTTATCGTCGGCGAAGTAGAA | RANDOM49 | GGAACTCAGGTGTCTATAGCGAG |
| RANDOM25 | CATCGGGTGGTGAACAAGGGTGA | RANDOM50 | TCGAGAGAGATTTCAAGACGCAT |
